# Unlocking the Functional and Nutritional Potential of Microalgae Proteins in Food Systems: A Narrative Review

**DOI:** 10.3390/foods14091524

**Published:** 2025-04-26

**Authors:** José A. M. Prates

**Affiliations:** 1 CIISA—Centro de Investigação Interdisciplinar em Sanidade Animal, Faculdade de Medicina Veterinária, Universidade de Lisboa, Av. da Universidade Técnica, 1300-477 Lisboa, Portugal; japrates@fmv.ulisboa.pt; 2 Associate Laboratory for Animal and Veterinary Sciences (AL4AnimalS), Av. da Universidade Técnica, 1300-477 Lisboa, Portugal

**Keywords:** microalgae proteins, protein extraction, functional properties, nutritional evaluation, food applications

## Abstract

As the global demand for sustainable, nutrient-rich protein sources intensifies, microalgae have emerged as a promising alternative due to their unique biochemical, environmental, and functional properties. This narrative review synthesises the nutritional value, protein composition, functional behaviour, processing technologies, and food applications of microalgae proteins. A literature search was conducted using PubMed, Scopus, and Web of Science, with keywords including “microalgae proteins”, “nutritional value”, “functional properties”, and “alternative protein sources”. Priority was given to peer-reviewed articles from the past decade that addressed nutritional quality, extraction methods, and food applications. Key species, Spirulina, *Chlorella*, *Nannochloropsis*, and *Haematococcus*, are highlighted for their high protein content (up to 70% dry weight), complete amino acid profiles, and rich bioactive compound content. Microalgae proteins show excellent solubility, emulsification, gelation, and foaming abilities, enabling use in dairy alternatives, baked goods, snacks, and 3D-printed foods. Advances in extraction, purification, and protein modification have improved their functionality, while cultivation on non-arable land and integration into circular biorefineries enhance sustainability. Remaining challenges include scalability, sensory optimisation, and regulatory clarity. Future studies should focus on improving sensory acceptance, optimising cost-effective processing, and expanding consumer awareness. Overall, microalgae proteins offer a robust and eco-efficient solution to meet global nutrition and sustainability goals.

## 1. Introduction

The global food system is under immense pressure to meet the nutritional needs of a growing population while reducing its environmental impact. With the world population expected to exceed 9 billion by 2050, protein demand is projected to rise by 32–78% compared to 2017 levels, placing extraordinary pressure on agricultural systems and natural resources [1]. Traditional animal-based protein production contributes significantly to environmental degradation, consuming extensive land, freshwater, and energy while emitting substantial amounts of greenhouse gases [2]. Livestock farming is also linked to deforestation, biodiversity loss, and nutrient pollution, raising critical questions about its long-term viability [3].

The search for sustainable, scalable, and health-promoting alternative protein sources has intensified in response to these concerns. One such promising avenue lies in exploring microalgae, with unicellular photosynthetic organisms offering high nutritional value, environmental benefits, and diverse food system applications [4,5].

Microalgae are microscopic, predominantly unicellular organisms that perform oxygenic photosynthesis using sunlight, carbon dioxide, and inorganic nutrients. Found in freshwater and marine environments, they are among Earth’s most ancient life forms and have evolved remarkable adaptability. Microalgae differ from higher plants in that they lack roots, stems, or leaves and typically exist as independent cells or simple colonies. Their photosynthetic efficiency and rapid biomass accumulation make them highly attractive for biotechnology and sustainable food production [6,7].

Microalgae are classified into several major groups taxonomically based on their cell structure, pigments, and storage products. These include cyanobacteria (also known as blue-green algae), which are prokaryotic and include species such as Spirulina (*Limnospira platensis*, formerly *Arthrospira platensis*); chlorophytes, or green algae, such as *Chlorella* and *Scenedesmus*, which are eukaryotic; diatoms (Bacillariophyta), which possess silica-based cell walls and include *Phaeodactylum tricornutum*; and red algae (Rhodophyta) like *Porphyridium*. Other groups include eustigmatophytes such as *Nannochloropsis* and flagellated species like *Tetraselmis* [8].

Microalgae have a very high protein content, with many species yielding between 40% and 70% protein by dry weight [5,9]. This exceeds or matches traditional protein-rich crops like soy and is on par with animal-derived sources. Importantly, microalgae proteins are nutritionally complete, containing all nine of the essential amino acids humans require. Their high digestibility and protein quality scores, such as the protein digestibility-corrected amino acid score (PDCAAS), further establish their suitability as a dietary protein source [10].

Beyond their macronutrient profile, microalgae are rich in bioactive compounds such as polyunsaturated fatty acids (including omega-3), vitamins (notably B12 and E), antioxidants, pigments like phycocyanin and astaxanthin, and essential minerals including iron and magnesium [5]. These compounds provide additional health benefits ranging from anti-inflammatory and antioxidant effects to cardiovascular and liver support [9,11].

Microalgae proteins also demonstrate valuable functional properties that enhance their versatility in food systems. These include excellent solubility across a wide pH range and strong emulsifying, foaming, and gelation capabilities. These traits enable their use in various food applications such as plant-based beverages, dairy analogues, baked goods, meat alternatives, snacks, and emerging formats like 3D-printed foods [12]. These functional properties are comparable to other plant-derived proteins used in dairy alternatives, such as rice bran protein, which has shown positive effects on the rheological and sensory characteristics of dairy desserts [13]. Their functionality also supports clean-label trends by reducing the need for artificial emulsifiers or stabilisers [14].

Environmentally, microalgae stand out for their low resource footprint. They can be cultivated on non-arable land using non-potable water, including saline or wastewater streams, thus avoiding direct competition with conventional food crops. Additionally, their photosynthetic growth process captures carbon dioxide, offering a pathway for carbon mitigation in integrated food-energy systems [15].

This narrative review aims to provide an in-depth and multidisciplinary examination of microalgae proteins, with a focus on their nutritional quality, functional properties, and versatility across various food applications. It investigates the biochemical and taxonomic diversity of microalgal species, evaluates state-of-the-art techniques for protein extraction and processing, and assesses the potential for integrating microalgae production into circular bioeconomy models to enhance sustainability.

## 2. Methods

To ensure a comprehensive and current overview, an extensive literature search was conducted using major scientific databases, including PubMed (National Centre for Biotechnology Information, Bethesda, MD, USA), Scopus (Elsevier, Amsterdam, The Netherlands), and Web of Science (Clarivate, London, UK). The search strategy targeted key terms such as “microalgae proteins”, “sustainable proteins”, “nutritional value”, “functional food ingredients”, “protein extraction”, “biorefinery”, and “alternative protein sources”. Priority was given to peer-reviewed studies published within the past decade to reflect recent advancements, identify existing research gaps, and highlight emerging challenges and opportunities for industrial-scale adoption of microalgae proteins in the global food system.

## 3. Integrated Perspectives on Microalgae Proteins

Microalgae proteins represent a unique intersection of biological diversity, nutritional excellence, and functional versatility. Understanding their role in food systems requires a holistic view of the compositional and structural characteristics that influence both their health benefits and processing behaviour. This section provides an integrated overview of the biochemical diversity among key microalgae species, their nutritional profiles, and the functional attributes that make them suitable for a wide range of food applications. By linking species-specific traits to performance outcomes, this perspective helps illuminate how microalgae proteins can be tailored for optimal use in modern, sustainable food systems.

### 3.1. Diversity and Biochemical Composition

Microalgae encompass a phylogenetically diverse group of unicellular, photosynthetic organisms that thrive in a wide variety of aquatic environments. This diversity includes prokaryotic cyanobacteria (such as Spirulina) and eukaryotic taxa like chlorophytes (e.g., *Chlorella vulgaris*), diatoms (e.g., *Phaeodactylum tricornutum*), and eustigmatophytes (e.g., *Nannochloropsis*) [8]. These microalgae differ widely in morphology, pigment content, growth characteristics, and metabolite production, enabling diverse applications in food and biotechnology.

Biochemically, many microalgal species exhibit high protein contents, ranging from 40% to 70% of dry biomass, making them highly competitive with conventional protein sources like soy or animal products [16]. The amino acid composition is often well-balanced, with many species providing all the essential amino acids necessary for human nutrition [17]. Genetic variability among species, along with external factors such as light intensity, salinity, pH, and nutrient availability, significantly influences protein yields and composition [18].

Furthermore, microalgae produce valuable bioactive compounds including polyunsaturated fatty acids, antioxidants (e.g., astaxanthin, β-carotene), vitamins (B12, E), and minerals (iron, magnesium), further increasing their appeal as functional ingredients [4,19]. For instance, *Haematococcus pluvialis* and *Dunaliella salina* are confirmed as the leading microalgae for the commercial production of astaxanthin and β-carotene, respectively, with astaxanthin reaching up to 7% and β-carotene up to 13% of dry weight [20].

### 3.2. Nutritional Quality and Health Benefits

The nutritional profile of microalgae proteins is increasingly well-documented. Proteins from species such as Spirulina and *Chlorella* exhibit high digestibility and excellent amino acid profiles, with PDCAAS and Digestible Indispensable Amino Acid Scores (DIAAS) often comparable to conventional protein sources [14]. These proteins support muscle synthesis, metabolic regulation, and tissue repair, making them ideal for use in general nutrition and sports recovery formulas. In addition to macronutrients, microalgae provide a rich profile of micronutrients and bioactive compounds. Spirulina is an exceptional source of iron and B-complex vitamins, especially of B12 in a form potentially bioavailable to humans [11], while *Chlorella* is noted for its chlorophyll and antioxidant contents [9]. Moreover, many microalgae contain omega-3 fatty acids like eicosapentaenoic acid (EPA) and docosahexaenoic acid (DHA), offering a plant-based alternative to fish oils [21].

Hydrolysis of microalgae proteins can release peptides with biological activities. *In vitro* and *in silico* studies have shown that peptides derived from *Chlorella* and Spirulina can act as inhibitors of digestive enzymes such as α-amylase and α-glucosidase, making them promising for managing blood glucose and metabolic syndrome [17]. Other studies suggest that these peptides also possess antihypertensive, anti-inflammatory, and immunomodulatory effects [22], indicating a strong potential for future functional food and nutraceutical development.

### 3.3. Functional Attributes in Food Systems

Equally important are the functional properties of microalgae proteins, which enhance their value as food ingredients. Their amphiphilic structure allows microalgae proteins to act as effective emulsifiers, foaming agents, and gelling agents across diverse pH and temperature conditions [23]. For instance, Spirulina-based proteins have been used in dairy-free spreads and beverages to provide smooth textures and enhance stability, while *Chlorella* proteins support structure and gelation in meat analogues and baked products.

Pigmented microalgae such as *Haematococcus* and *Dunaliella* also provide natural colourants (e.g., astaxanthin and β-carotene), which can improve product aesthetics and support clean-label formulation strategies [24]. The multifunctional role of microalgae, providing both nutritional enhancement and techno-functional performance, streamlines ingredient lists and meets growing consumer preferences for natural, minimally processed foods.

Moreover, microalgae show promise in improving the sensory and structural attributes of food. In gluten-free bread, the inclusion of microalgae like *Nannochloropsis* and *Chlamydomonas* improved the protein content and textural quality while increasing micronutrient density and antioxidant capacity [25]. These functional advantages open new doors for microalgae in baking, dairy substitutes, snacks, and meat analogues.

Table 1 consolidates data across major microalgae species used in food systems. It compares protein content, essential amino acid balance, presence of bioactive compounds, and functional traits such as solubility, emulsification, and gelation. For instance, Spirulina is characterised by a high protein content and excellent foaming capacity, while *Chlorella vulgaris* provides a well-rounded amino acid profile and gelation support. *Nannochloropsis* offers a strong profile in omega-3 fatty acids, whereas *Haematococcus pluvialis* is a source of the potent antioxidant astaxanthin. This integration serves as a practical tool for food technologists and researchers, enabling the rapid assessment of microalgae species for specific product applications. Importantly, cultivation parameters and downstream processing techniques can be fine-tuned to further enhance desired traits such as pigment concentration or peptide bioactivity [26].

## 4. Integrated Processing Strategies: Extraction, Purification, and Protein Modification

The development of microalgae-based protein ingredients relies heavily on efficient, integrated processing strategies that span from initial cell disruption to downstream purification and functional tailoring. These interconnected steps, extraction, purification, and modification, not only determine the final yield but also define the structural, nutritional, and functional characteristics of the recovered proteins. A systematic approach that preserves protein integrity while maximising performance is crucial to position microalgae as viable alternatives to traditional protein sources in food systems.

### 4.1. Extraction Techniques and Cell Disruption

Microalgal proteins are located intracellularly, necessitating effective cell wall disruption for extraction. The complexity and rigidity of microalgae cell walls vary across species, influencing the required extraction technique. For example, *Chlorella vulgaris* has a particularly tough, cellulose-rich wall, while Spirulina lacks a conventional cellulose wall, allowing for easier extraction [70].

Mechanical disruption methods remain among the most widely used for breaking these barriers. High-pressure homogenization subjects microalgal biomass to extreme shear forces, rupturing cell walls but potentially denaturing sensitive proteins due to friction-induced heat. Ultrasonication, another common method, employs high-frequency sound waves to generate cavitation bubbles that physically disrupt cells. Although it reduces thermal damage compared to homogenization, it requires careful energy control to prevent localised overheating [71].

Emerging non-thermal approaches like pulsed electric field (PEF) treatment offer a more protein-friendly solution. PEF applies short high-voltage bursts to induce electroporation, forming temporary pores in the cell membrane. This allows intracellular proteins to diffuse out with minimal structural degradation, particularly useful for preserving heat-sensitive fractions [72].

Enzymatic lysis represents a complementary or alternative approach. By targeting specific cell wall components using cellulases, proteases, or lysozymes, enzymatic treatments offer a selective and mild strategy to extract proteins while minimising energy input and protein denaturation [73,74]. When paired with mechanical disruption, enzymatic treatment enhances extraction efficiency and protein integrity [75,76].

### 4.2. Purification Strategies

Following extraction, purification is essential to isolate the protein fraction and remove unwanted components such as pigments, lipids, and carbohydrates. One of the most accessible methods is isoelectric precipitation, where proteins are precipitated at their isoelectric point (pI), the pH at which their net charge is zero. This technique allows for bulk protein recovery, especially in species with well-characterised pI values [77].

Membrane-based technologies such as ultrafiltration and diafiltration are widely used in continuous production environments. These techniques separate molecules based on size, concentrating proteins while removing small molecular weight impurities like salts or pigments. Their scalability and chemical-free nature make them attractive for food-grade applications [78].

For applications requiring highly pure protein fractions, chromatographic methods such as ion exchange, affinity, and size-exclusion chromatography can be employed. These are particularly effective for isolating bioactive proteins, like phycocyanin from Spirulina, or low-molecular-weight peptides with specific functionalities [75]. However, their resource and cost intensity may limit industrial-scale adoption unless integrated efficiently.

### 4.3. Protein Modification Methods

Once isolated, proteins can be further modified to enhance functional properties such as solubility, emulsification, foaming, or bioactivity. Among these, enzymatic hydrolysis is one of the most promising. Controlled treatment with proteases like Alcalase or Trypsin cleaves specific peptide bonds, reducing molecular weight and exposing functional groups. These modifications increase digestibility and often produce peptides with antioxidant or antihypertensive activity [62].

For example, hydrolysates from *Scenedesmus obliquus* prepared using optimised Alcalase–Trypsin treatment showed an excellent emulsifying and fat-binding capacity, especially for peptides under 10 kDa [62]. Similarly, *Chlorella*-derived peptides under 3 kDa produced using ultrasound-assisted enzymatic hydrolysis demonstrated both ACE-inhibitory and antioxidant activity, highlighting their dual nutritional and functional roles [79].

In addition to hydrolysis, pH shifting (exposing proteins to extreme acidic or alkaline conditions) can be used to unfold and refold protein structures, enhancing solubility and gelation. Thermal treatments, if carefully controlled, can expose hydrophobic regions that improve the emulsifying capacity without causing aggregation or loss of functionality [77].

Cross-linking techniques, whether enzymatic (e.g., transglutaminase) or physical (e.g., high-pressure processing), can further tailor the texture and network-forming abilities of proteins. This is particularly useful in applications such as plant-based meat or dairy analogues, where gelling and water retention are critical [75].

### 4.4. Linking Processing to Functionality

Each processing step, from cell disruption to final modification, directly impacts the techno-functional and nutritional properties of the protein product. Overly aggressive mechanical treatment may yield high protein recovery but result in denaturation that reduces solubility and bioactivity. In contrast, a mild enzymatic treatment may produce a lower yield but result in peptides with superior emulsification, digestibility, and health benefits [71].

The ability to selectively enhance certain functionalities, such as improving foaming for whipped toppings or boosting solubility for beverages, through controlled processing is one of the key advantages of microalgal proteins. This flexibility supports their use in a wide variety of formulations, from sports nutrition to dairy alternatives, clean-label sauces, and even functional snacks [62].

In summary, the ability to integrate and optimise cell disruption, extraction, purification, and protein modification techniques is vital to unlocking the potential of microalgae as sustainable protein sources. These strategies must strike a careful balance between maximising recovery and preserving the molecular characteristics responsible for desirable nutritional and functional properties. Continued innovations in non-thermal, enzymatic, and eco-friendly approaches will be essential in making microalgae proteins competitive in the growing market for alternative and functional protein ingredients.

## 5. Applications of Microalgae Proteins in Food Systems

Microalgae proteins are emerging as a versatile and sustainable ingredient across a wide range of food sectors, thanks to their high nutritional value, functional properties, and environmentally friendly production. Their solubility, emulsification, foaming, and gelling capacities make them particularly well-suited for incorporation into dairy alternatives, baked goods, extruded snacks, beverages, and even 3D-printed foods. These characteristics also support the rising consumer demand for plant-based, clean-label, and nutritionally dense food products.

### 5.1. Dairy Alternatives and Fermented Products

Microalgae proteins hold significant potential as functional ingredients in the development of dairy alternatives such as plant-based yoghurts, milks, cheeses, and probiotic beverages. Their complete amino acid profiles, high digestibility, and excellent emulsifying and water-holding capacities allow them to replicate the structural and textural roles of bovine milk proteins, making them well-suited for dairy-mimetic formulations [80]. For example, proteins from species like Spirulina and *Chlorella* have been shown to stabilise emulsions and improve mouthfeel in plant-based milks, while also contributing valuable micronutrients such as vitamin B12 and iron [21].

In fermented dairy alternatives, microalgae proteins enhance both functional and nutritional attributes. *Chlorella vulgaris* and *Pavlova lutheri*, when incorporated into yoghurt-like products, have been reported to boost antioxidant activity, increase mineral bioavailability (e.g., iron and magnesium), and positively influence sensory qualities such as colour and flavour [81]. In addition, fermentation may improve the release and bioaccessibility of microalgae-derived nutrients, contributing to enhanced functional properties [18].

Recent studies also suggest that the fermentation of microalgae-enriched matrices can enhance protein digestibility and release bioactive peptides with antihypertensive or antioxidant activity, supporting their role in functional fermented foods [22]. These multifunctional traits make microalgae proteins promising candidates for formulating the next generation of clean-label, plant-based dairy alternatives tailored for both health benefits and consumer acceptance.

### 5.2. Bakery, Pasta, and Extruded Snacks

The bakery industry is another area where microalgae proteins are being actively explored. For instance, *Chlorella vulgaris* has been used to replace 100% of animal-based fat and protein in brioche-type baked goods without compromising texture or flavour, and in some cases improving them [82]. Microalgae proteins enhance dough elasticity, improve moisture retention, and extend shelf life, making them a valuable functional addition in both traditional and gluten-free formulations.

In pasta and noodles, the incorporation of microalgae proteins contributes not only to the protein content but also to cooking stability and firmness. Fortified pasta enriched with Spirulina or *Chlorella* exhibits higher levels of micronutrients such as iron and vitamin B12, and retains an appealing texture during cooking [6].

Extruded snack products also benefit from the structure-forming and water-binding abilities of microalgae proteins. Their integration into cereals, crackers, and chips results in snacks with enhanced crispness and protein density, while also contributing antioxidant and anti-inflammatory benefits [83].

### 5.3. Emerging Food Formats and Innovative Applications

One of the most exciting areas of application is 3D food printing, where rheological control and structural stability are critical. Microalgae proteins enhance batter printability, helping maintain shape fidelity and texture in extrusion-based printing systems. Research has shown that snacks enriched with 3–5% *Chlorella* or Spirulina maintain structural accuracy and show improved nutritional value [84].

Although higher concentrations may affect sensory appeal due to strong pigmentation, emerging technologies such as coaxial printing are being explored to mask colour while preserving nutritional value. This opens pathways for personalised nutrition, where microalgae can be tailored to create meals that target specific dietary needs.

Microalgae proteins are also being used in functional beverages and plant-based smoothies. Their high solubility and complete amino acid content make them ideal for sports recovery drinks, senior nutrition supplements, and high-performance wellness beverages. When combined with fruits or plant milks, microalgae proteins contribute not only to protein content but also to antioxidant capacity and micronutrient delivery [18].

### 5.4. Functional Foods and Nutraceuticals

Beyond conventional food applications, microalgae proteins are increasingly incorporated into functional foods and nutraceutical products. Enzymatic hydrolysis of these proteins yields bioactive peptides with significant health benefits, including antihypertensive, antioxidant, anti-inflammatory, and cholesterol-lowering effects [85]. These peptides can be used in dietary supplements and fortified foods aimed at managing chronic diseases, particularly cardiovascular conditions.

Peptides derived from Spirulina and *Chlorella* have demonstrated ACE-inhibiting activity, a target in hypertension management. Their integration into capsule, powder, or beverage forms enables flexible delivery platforms in health and wellness markets [10].

### 5.5. Clean-Label Colourants and Aesthetic Enhancements

Many species of microalgae, such as *Haematococcus pluvialis* and *Dunaliella salina*, produce highly pigmented compounds like astaxanthin and β-carotene. These pigments serve dual purposes: they act as natural antioxidants and provide a vibrant colour that enhances product appeal [24]. With the rising consumer demand for clean-label products, replacing synthetic dyes with microalgae-derived pigments offers a valuable marketing and health advantage.

Applications include natural food colourants in beverages, confections, yoghurts, and sauces. The added nutritional benefits make them a multifunctional solution for food developers looking to combine aesthetics and health.

The integration of microalgae proteins into food systems spans both traditional and emerging formats, demonstrating their multifunctionality and future potential (see Table 1). From improving dough structure in baked goods to supporting muscle recovery in drinks, or even building structural frameworks in 3D-printed snacks, microalgae proteins are pushing the boundaries of food innovation. Their clean-label credentials, functional health benefits, and environmental sustainability make them well-positioned to play a major role in the next generation of food products. Continued advances in cultivation, processing, and sensory optimisation will further expand their utility across mainstream and specialised food markets.

**Figure 1:**
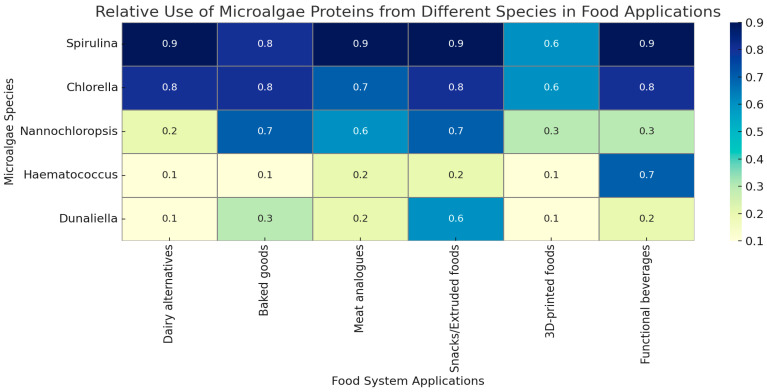
Relative use of proteins from selected microalgae species in different food system applications. The heatmap values (0–1) represent an estimated proportion of usage based on their functionality, nutritional profile, and prevalence in food product development literature. Data from [10,18,21,80,81,82,83,84,85].

Figure 1 illustrates the relative extent to which key microalgae species are utilised in various food applications, including dairy alternatives, baked goods, meat analogues, snacks, functional beverages, and 3D-printed foods. These proportions reflect both reported functional suitability and prevalence in the literature.

## 6. Sustainability, Scalability, and Economic Viability

The long-term success of microalgae proteins in the global food sector depends not only on their nutritional and functional merits but also on their environmental footprint, production scalability, and economic feasibility. This section explores the multifaceted sustainability benefits of microalgae cultivation, including low land and water requirements, carbon capture potential, and integration with circular resource systems. It also examines the operational challenges associated with large-scale production, from cultivation system limitations to biomass variability, and highlights the critical role of integrated biorefinery models in improving cost-efficiency. Together, these perspectives provide a comprehensive assessment of the practical and economic factors shaping the future adoption of microalgae proteins in sustainable food systems.

### 6.1. Environmental Sustainability and Resource Efficiency

Microalgae are widely recognised as one of the most promising sustainable protein sources due to their low land footprint, high productivity, and capacity to thrive in conditions unsuitable for conventional agriculture. They can be cultivated on non-arable or marginal lands using saline or brackish water, significantly reducing dependence on freshwater and eliminating competition with food crops [86].

Microalgae also offer substantial environmental benefits through their rapid growth cycles and high photosynthetic efficiency, allowing for exceptional areal productivity. Many species double their biomass within 24–48 h, far outpacing terrestrial crops in growth rates and yield per hectare [87]. Moreover, their photosynthetic mechanism enables the direct capture and conversion of carbon dioxide into biomass, contributing to greenhouse gas mitigation and aligning with global decarbonization goals [88].

An especially impactful application lies in coupling microalgae cultivation with wastewater treatment. Industrial and municipal effluents, rich in nitrogen and phosphorus, can be repurposed as nutrient sources, thus eliminating the need for synthetic fertilisers while reducing the ecological burden of wastewater discharge [89]. This approach turns waste into a valuable input, exemplifying circular bioeconomy principles and supporting closed-loop production models [90]. However, it is important to note that the use of biomass grown on such substrates for human or animal consumption is subject to strict regulatory scrutiny. While promising from an environmental standpoint, such applications currently face significant legislative hurdles and are typically limited to non-food uses unless thorough safety validation is performed.

Additionally, microalgae cultivation integrates well with flue gas recycling from industrial sources, providing a dual benefit of carbon sequestration and biomass generation. Some pilot projects have demonstrated reductions in net CO_2_ emissions of over 50% when flue gases are used as a carbon input for microalgal growth [91].

### 6.2. Technical and Operational Challenges in Scaling

Despite their potential, the transition from laboratory-scale to commercial-scale microalgae production is fraught with technical and operational challenges. Open raceway ponds, though cost-effective, are highly susceptible to contamination, evaporation, and environmental fluctuations that can disrupt growth and reduce protein yields. They also offer limited control over nutrient delivery and pH stabilisation, which are essential for producing consistent biomass quality [87].

In contrast, closed photobioreactors (PBRs) provide better control over temperature, CO₂ levels, and contamination, resulting in higher biomass and protein quality. However, PBRs involve significantly higher capital and operating costs, often requiring energy-intensive lighting, mixing, and CO_2_ regulation. Hybrid systems, combining the scalability of open ponds with the precision of PBRs, are being explored as a potential compromise [92].

Another critical barrier is the harvesting process. Techniques such as centrifugation are effective but energy-intensive and costly, while flocculation, though more affordable, raises concerns regarding the safety of chemical additives for food or feed applications. Regulatory frameworks may restrict the use of certain flocculants unless proven to be food-grade or used in closed-loop systems. As the reviewer noted, biomass produced via fermentation may offer a more controlled and compliant route for human and animal nutrition. These factors must be carefully considered when designing scalable and compliant harvesting strategies.

One major bottleneck in scalability is the biomass variability resulting from external factors such as light intensity, nutrient availability, and water chemistry. This inconsistency affects downstream processes like extraction, purification, and formulation. To mitigate this, real-time monitoring technologies using machine learning, sensor networks, and automated feedback systems are being adopted to ensure optimal growth conditions and standardise output quality [91].

Furthermore, genetic engineering and strain improvement strategies hold promise for enhancing protein yields and resistance to environmental stressors. Selective breeding and CRISPR-based editing can lead to strains with improved uniformity, enabling more predictable scaling and efficient downstream processing [86].

### 6.3. Economic Considerations and Integrated Biorefinery Approaches

Achieving economic viability remains one of the biggest hurdles for commercial microalgae protein production. High production and processing costs continue to limit scalability, especially when microalgae are treated as single-output systems. As a solution, the integrated biorefinery model is gaining traction, an approach that enables the recovery of multiple co-products from a single biomass input.

For example, while protein may be the primary target, high-value pigments like phycocyanin from Spirulina and astaxanthin from *Haematococcus* can generate substantial revenue in the nutraceutical and cosmetic markets [24]. Lipid fractions containing omega-3 fatty acids can also be purified for food and feed, while residual biomass may be converted into biofertilizers or biochar, completing the resource loop [89].

Integrating renewable energy sources such as solar thermal or photovoltaic systems into cultivation operations can significantly lower energy inputs. Automation, such as AI-based light modulation or nutrient dosing, also reduces labour and enhances reproducibility, contributing to long-term economic resilience [90].

Finally, regulatory frameworks and market acceptance play a crucial role in economic viability. Harmonised safety and labelling standards will reduce barriers to entry, while educational campaigns about the nutritional and ecological benefits of microalgae can accelerate consumer uptake, especially in high-growth markets for plant-based and functional foods [85]. For example, Spirulina and *Tetraselmis chui* have received Generally Recognised As Safe (GRAS) status in the US and Novel Food approval in the EU.

Microalgae proteins represent a promising, sustainable, and innovative solution for the global protein crisis. Their potential to be cultivated on non-arable land using waste streams while simultaneously capturing carbon dioxide places them at the forefront of environmentally responsible food technologies. However, achieving large-scale adoption requires addressing several technical and economic bottlenecks. Integrated biorefineries, automation, genetic optimisation, and hybrid cultivation systems are paving the way toward commercial viability.

## 7. Conclusions and Future Perspectives

Microalgae proteins offer a unique combination of nutritional, functional, and environmental benefits that position them as a strong alternative to conventional protein sources. They provide complete amino acid profiles, high digestibility, and are rich in bioactive compounds such as antioxidants and polyunsaturated fatty acids. Functionally, microalgae proteins exhibit excellent solubility, emulsification, and gelation properties, enabling their use in diverse food systems, including dairy alternatives, baked goods, and innovative formats like 3D-printed foods. From an environmental standpoint, microalgae require non-arable land and can thrive using saline or wastewater, significantly reducing pressure on freshwater and agricultural resources. Their ability to capture carbon dioxide during photosynthesis further supports climate change mitigation and sustainable food production models.

Despite these advantages, several challenges remain. The scaling-up of cultivation systems is hindered by variability in biomass quality due to fluctuating environmental conditions. Real-time monitoring and automation are essential to ensuring consistent production. Extraction and purification methods also need refinement, particularly for species with tough cell walls. Improvements in enzymatic disruption and membrane technologies will be key to enhancing protein recovery efficiency, preserving bioactive properties, and enabling the cost-effective scale-up of microalgae protein production for food applications. Sensory issues, such as strong flavours and pigmentation, limit consumer acceptance. Research into depigmentation, flavour masking, and protein modification is crucial to enhance the palatability of microalgae-derived products without compromising nutritional value.

Economic viability depends on integrated biorefinery approaches that allow for the co-extraction of high-value compounds, such as pigments and lipids, alongside proteins. These diversified revenue streams can help offset production costs. Collaboration between industry and academia is critical to developing cost-effective, scalable technologies. Looking ahead, microalgae proteins are poised to play an important role in future food systems. Advances in genetic engineering and selective breeding will help improve protein content, simplify extraction, and reduce sensory barriers. Interdisciplinary research spanning biology, food science, engineering, and consumer studies will be key to overcoming current limitations. Wider adoption will also require clearer regulatory frameworks and greater consumer awareness. Transparent standards and education around the health and sustainability benefits of microalgae proteins can foster trust and accelerate market growth.

## Figures and Tables

**Table 1:** Overview of proteins and extracts derived from various microalgal species, highlighting their key characteristics and potential applications in food systems. Abbreviations: AA, amino acid; dw, dry weight; FAO, Food and Agriculture Organisation; GRAS, Generally Recognized As Safe; PDCAAS, Protein Digestibility Corrected Amino Acid Score.

Microalgae Species	Protein/Extract Name	Physical Properties	Chemical Properties	Nutritional Properties	Food Applications	References
*Aphanizomenon flos-aquae*	Whole microalga protein (contains C-phycocyanin pigment)	Water-dispersible cyanobacterial biomass; similar functional traits to Spirulina (good water solubility, contributes blue colour); moderate stability (must monitor microcystin toxin contamination)	Rich in phycobiliproteins (e.g., C-phycocyanin ~α: 17 kDa, β: 18 kDa subunits); ~60–70% protein content (dry weight)	Complete AA profile (all essential AAs present); high protein (60–70% dw) with all 20 standard AAs; essential AAs-rich (score ~1.0) and contains bioactive compounds (e.g., phenylethylamine)	Dietary supplements (dried algae powders, tablets); added to smoothies and health foods for protein and micronutrients; historical use as food (e.g., harvested from Klamath Lake)	[27,28,29]
*Auxenochlorella protothecoides* (syn. *Chlorella protothecoides*)	Whole Algal Protein, heterotrophic *Chlorella* protein isolate	Extremely high solubility across a broad pH 2–12 (84–100% protein solubility); forms stable emulsions (oil-in-water) up to 7–14 days, outperforming whey protein; emulsions remain stable in high salt (0.5 M) and pH 2–9	Predominantly polar, hydrophilic AAs in its proteins; many proteins are glycosylated, contributing a negative charge and water-binding; isoelectric precipitation yields pI ~4–5 for major fractions	Protein >60% dw; provides all essential AAs (high lysine, leucine); essential AA index >1.0 (exceeds FAO requirements); digestibility improved by cell disruption	Approved as novel protein ingredient (Canada, EU); used in protein supplements and blended into baked goods, beverages, and sauces for its neutral colour and high protein; studied in vegan mayonnaise and dressings	[30,31,32]
*Chlamydomonas reinhardtii*	Chlamydomonas biomass protein (rich in RuBisCO enzyme)	Green algal cells (motile) with protein largely in soluble form; moderate water solubility when cell is lysed; no known specialised extract; functional properties presumed similar to other chlorophytes	RuBisCO (ribulose-1,5-bisphosphate carboxylase) is ~560 kDa complex; protein ~48% dw in *C. reinhardtii*; balanced AA profile	Protein ~48% dw; complete AA profile (meets FAO requirements); high nutritive value and considered GRAS for use in foods	Emerging as a novel food ingredient; potential for use in nutrient-rich shakes, fermented foods, and as a host to produce recombinant proteins in edible form	[33,34,35]
*Chlorella vulgaris*	*Chlorella* protein (algal protein isolate or concentrate)	Fine green powder (cell-wall ruptured) disperses in water; solubility is lowest around pH ~4–5, highest at pH 7–8; good emulsifying capacity	Protein ~50–60% dw; contains RuBisCO (large subunit ~55 kDa) and light-harvesting chlorophyll-binding proteins (~20–30 kDa); all essential AAs present	High-quality protein with balanced profile (all 9 essential AAs); digestibility ~70–80% (cell wall hinders full digestibility)	Commercially popular in supplements (tablets, powders); incorporated into pastas, crackers, breads, and beverages for protein fortification	[36,37,38]
*Dunaliella bardawil*	*Dunaliella* protein (from β-carotene-rich algae)	Lacks a rigid cell wall; moderate water solubility; stability influenced by the high salt environment of growth	Protein ~10–35% dw; complete essential AA profile (AA score ~1.06)	Protein ~10–35% dw; all essential AAs present in balanced proportions	Used mainly as a natural β-carotene source in supplements and food colourant; whole biomass can enrich foods with protein	[39,40,41]
*Dunaliella salina*	*Dunaliella* protein (algal biomass protein)	No cellulosic cell wall, so cells are easily ruptured: proteins are water-soluble and accessible	Protein content ranges from ~20% up to 57% dw depending on growth conditions	Protein ~20–57% dw; rich in essential AAs; good digestibility due to lack of a hard wall	Primarily used for β-carotene (as a natural colourant and supplement); dried biomass has been added to specialty foods	[40,42,43]
*Euglena gracilis*	*Euglena* protein (from *Euglena* biomass)	Grown heterotrophically or photoautotrophically; fairly soluble proteins; mild extraction yields light beige powder	Notable for high content of sulphur AAs (cysteine, methionine); contains all 20 AAs	Protein ~30–39% dw; well-balanced essential AA profile; highly digestible	Incorporated into health foods and supplements; used in protein shakes and bars	[44,45,46]
*Haematococcus pluvialis*	*Haematococcus* protein (from astaxanthin-rich alga)	High water-holding capacity; good emulsifier; exhibits excellent foaming	Proteins include stress-related enzymes and RuBisCO; protein ~30–45% dw	Protein ~40% dw; high-quality AA profile; digestibility ~80% after cell breakage	Used commercially for astaxanthin; the remaining protein-rich meal is studied for use in foods	[47,48,49]
*Nannochloropsis gaditana*	Nannochloropsis protein (algal protein concentrate or hydrolysate)	Good emulsifying capacity; stable foams; moderate solubility	Protein ~19% to 45% dw depending on strain and growth; balanced AA profile	Protein ~20–45% dw; rich in glutamate and aspartate; good digestibility	Gaining approval as a novel food ingredient; used in breads and crackers	[50,51,52]
*Nostoc flagelliforme*	*Nostoc* protein (edible cyanobacterial biomass)	Forms filamentous colonies; physical functionality as isolated protein not well-studied	Moderate protein content (est. 20–30% dw); complete AA profile	Protein ~20–30% dw; all essential AAs present; digestibility presumed good when cooked	Eaten as a culinary ingredient in East Asia for centuries; modern use is limited due to overharvesting	[53,54,55]
*Phaeodactylum tricornutum*	*Phaeodactylum* protein (diatom biomass protein)	Silica frustule encases cells, needs disruption; moderate solubility	Lower protein content (~15–25% dw); complete but slightly limited in lysine and tryptophan	Protein ~15–20% dw; decent essential AA profile; digestibility ~70%	Incorporated into experimental foods for omega-3 and protein content	[56,57,58]
*Porphyridium cruentum*	R-Phycoerythrin (red protein pigment from *Porphyridium*)	Highly water-soluble; exhibits fluorescence; soluble across pH ~5–8	Multimeric protein (~240 kDa as (αβ)_6 complex); moderate protein content (~25–30% dw)	Protein ~25% dw; complete AA profile; low digestibility due to polysaccharide matrix	Used as a natural red food colourant and antioxidant protein	[59,60,61]
*Scenedesmus obliquus*	*Scenedesmus* protein (algal protein from *Scenedesmus*)	Good water-holding and gelation ability; excellent emulsifying properties	Protein ~50–60% dw; balanced AA profile	Protein ~50–56% dw; complete essential AA profile; digestibility improves with cell wall breakage	Used in bread, pasta, and snack crackers; promising for meat analogues	[62,63,64]
Spirulina (*Limnospira platensis*, formerly *Arthrospira platensis*)	Spirulina protein (e.g., C-phycocyanin; Spirulina protein isolate)	Highly soluble in water except near pI (~pH 3.5); thermal stability up to ~70 °C for short times; excellent emulsifier and foaming agent at neutral–alkaline pH	Isoelectric point ~3.5; major proteins are phycobiliproteins and RuBisCO; phycocyanin pigment (blue) is ~30–40 kDa per subunit, forming ~210 kDa complexes; low in sulphur AAs (methionine, cysteine)	Protein ~50–70% dw; complete protein with ~51–71% of AAs as essential; very digestible (80–90% digestibility); PDCAAS ~0.81	Widely used in foods and beverages: smoothies, nutritional bars, pasta, crackers; meat analogues and dairy-free cheese for protein fortification; C-phycocyanin from Spirulina is a natural blue colourant used in confections and drinks	[11,65,66]
*Tetraselmis chui*	*Tetraselmis* protein (algal protein from *Tetraselmis*)	Moderate solubility; good foaming and emulsifying capacity	Protein ~30–40% dw; high lysine and threonine content	Protein ~35% dw; complete essential AA profile; good digestibility	Approved as a novel food ingredient; used in sauces, seasonings, and protein shakes	[67,68,69]

## Data Availability

No new data were created or analysed in this study. Data sharing is not applicable to this article.
